# Stakeholder views on young adults with intellectual disabilities as a workforce: A qualitative study on students’ performance in upper secondary education and their employment potential

**DOI:** 10.1177/17446295211026475

**Published:** 2021-07-28

**Authors:** Kateryna Karhina, Jens Ineland, Lotta Vikström

**Affiliations:** Umeå University, Sweden; Umeå University, Sweden; Umeå University, Sweden

**Keywords:** upper-secondary education, employment opportunities, school-to-work transition, young adults with intellectual disabilities, special education

## Abstract

People with intellectual disabilities are the most disadvantaged group among all disability types when it comes to employment. In Sweden, special needs upper secondary schools prepare students with intellectual disabilities for the labour market using practice periods at workplaces. This study targets stakeholder involved in their school-to-work transition (i.e. teachers, employers, employment agency officials). The aim is to identify how they view: (1) the working capabilities of students during practice periods and (2) their employment potential. We base the analysis on interview data with the stakeholders using Grounded Theory. Our results identify three student types whose preparedness for the labour market differs considerably. One student type performs well during the practice period and represents a high potential to enter the workforce. The other two student types have the lower working capability and employment potential. Our study highlights stakeholders as resources to improve the labour market preparations of students with intellectual disabilities.

## Introduction

In contemporary society, paid employment is commonly viewed as a sign of promoting independence. It serves as a primary indicator of successful integration into society and a social marker for citizenship. Studies find that employment holds several positive outcomes for people’s psychological wellbeing and their material standard (e.g. Butler et al., 2016; Ellenkamp et al., 2016; Garcia-Villamisar and Hudges, 2007; Jahoda et al., 2008; Kocman et al., 2018). However, people with intellectual disabilities face more barriers in finding and maintaining paid employment compared to those without disabilities (Eisenman, 2003; Holwerda et al., 2015; Park and Park, 2019). This indicates there is a mismatch between the working skills and competence they present and can supply to the workforce, and the demands and expectations from employers in the labour market. Research has primarily focused on the persons with disabilities themselves (their needs, skills, motivation, etc.) in seeking to understand this employee-workplace mismatch, while explanatory factors in the surrounding context have been under-researched (Araten-Bergman, 2016).

Sweden is considered to be in the top rank for equality indices worldwide and has very high social expenditure. Nevertheless, people with disabilities and especially with intellectual disabilities have limited access to education, employment, social activities, political representation, and material resources compared to non-disabled citizens (Arvidsson et al., 2015; Lindberg, 2016; National Board of Health and Welfare Report, 2009, 2010; Tideman et al., 2017, 2020). The Swedish education system seeks to promote the transition to the labour market for young adults with disabilities. Special needs upper secondary schools (special needs USS) represent an independent form of schooling targeted towards students unable to meet the requirements of upper secondary school because of an intellectual disability. The primary goal for the special needs USS is to provide each student the opportunity to prepare for establishment in the labour market according to the National Agency of Education. To meet this goal, Workplace-Based-Learning (WBL) has been introduced to improve school-to-work transitions and increase the students’ chances of getting a job. The basic idea with WBL is to extend the students’ opportunities to put theory into practice with work experiences, so as to explore within a ‘real-world’ context what they have learned in the classroom. WBL provides students with the opportunity to spend a minimum of 22 weeks of education at workplaces with an assigned supervisor (Ineland et al., 2021). A student’s placement is primarily based on a learning plan with aims and objectives, which the student and supervisors at workplaces and the school, the latter being the most responsible for observing and assessing the student’s progress, have agreed to.

These types of schools and their practice periods constitute the context of our study. Previous research on school-to-work transitions has primarily focused on the preparation of students with intellectual disabilities and to what extent they meet labour market demands. Less is known about the roles and attitudes of stakeholders who are to act as resources for them during their education or afterwards to help them enter the workforce (Cavanagh et al., 2017; Dwertmann, 2016). As shown in research (Hernandez et al., 2000; Ineland et al., 2021), their difficulties in finding and maintaining employment are often coupled with stigmatisation and prejudices of employers and other stakeholders concerning intellectual disabilities. This notion calls us to focus on the stakeholders to increase the understanding of the mismatch between the working skills and competence people with intellectual disabilities represent and the demands and expectations from employers in the labour market.

Research has addressed multiple perspectives to explain and resolve this mismatch. Two concepts commonly used in disability research to understand the causes and needs for support of people with disabilities are the medical/individual model and the social/relational model of disability (McKenzie, 2013; Oliver, 2013). The former model refers to various limits in respect of the person’s bodily function which, in our study, would translate to diagnoses in students’ cognitive ability regarding language, social competence, and managing of expectations at a workplace. In contrast, the social/relational model emphasises contextual circumstances and conceptualises disability (and need for support) as a consequence of a gap between individuals’ preconditions and capability on the one hand, and various demands and expectations in society on the other hand (Oliver, 2013; Tøssebro and Söderström, 2010). Thus, disability is not primarily individually determined but results from more or less disabling environments. This view on disability calls for contextual changes, e.g. how a given situation and environment can be adjusted to an individual’s specific needs. Our study is theoretically based on a social/relational understanding of disability, which means that we acknowledge that low participation in the labour market among people with intellectual disabilities is closely linked to an individual-environmental mismatch (cf. Ballo, 2020; McKenzie, 2013). Our study reveals what these environmental circumstances look like for Swedish students transitioning to the labour market from special needs USS, as this would explain why young adults with intellectual disabilities are facing major barriers to becoming recognised as part of the workforce. To obtain information on this issue, we targeted three groups of stakeholders made up of teachers, placement employers, and employment agency officials.

### Aims of the study

Drawing on an ideal type approach, with an ambition to differentiate properties that characterise people with intellectual disabilities as workforce, the current paper aims to identify stakeholder views on (1) the students’ working capabilities during education and (2) their future employment potential. Focusing on ideal types, not individual students, constructed by consistent traits of students with intellectual disabilities, as they appear in the interview data, provides an analytical opportunity to bring together experiences from the different external positions and roles, which all have key roles in school-to-work transitions.

### Research overview

Research on stakeholders involved in the process of preparing students with intellectual disabilities for the labour market exists, but on a comparatively limited scale. We account for some key studies below, a few of which distinguish between different roles and responsibilities of certain stakeholders. Araten-Bergman (2016) argues that research on the demand-side such as social context and employers’ motivation and views are important to scrutinise to understand and enhance the employment opportunities for people with intellectual disabilities. Ellenkamp et al. (2016) argue that the attitudes of employers are a major factor for people with intellectual disabilities in entering the workforce. This indicates, as pointed out by Bachrach (2015), that vocational rehabilitation per se and job development strategies that focus solely on the individual with a disability are insufficient to meet the employment needs of people with disabilities. Moore and colleagues (2018) show that high-efficiency demands among employers often represent a barrier for employing people with disabilities. Another study (Kocman et al., 2018) concludes that when potential employers receive information on intellectual disability and the working capability of people with intellectual disabilities, for instance with best practice examples and role models, this increased the employers’ interest in hiring them. Some studies have also shown that having previous experiences from employees with intellectual disabilities makes employers more positive towards them as a workforce than employers without such experiences. This suggests that previous personal encounters can enhance employment opportunities (cf. Luecking, 2008, 2011; Morgan and Alexander, 2005). Hernandez and colleagues (2000) found mixed attitudes among employers, however, as there was an evident discrepancy between those expressing a willingness to hire people with intellectual disabilities and actually hiring them. Studies that are more recent report similar diverging opinions about disability and employment potential (Ineland et al., 2021; Vornholt et al., 2013; Zappella, 2015). The findings presented above stress the need to consider stakeholders’ views to understand how multiple factors beyond the students with intellectual disabilities themselves act as facilitators or barriers in their school-to-work transition.

## Data and methods

### Data collection and sampling of research participants

Our study concerns the northern part of Sweden and three stakeholder groups involved in special needs USS education or students’ subsequent transition to paid employment. The stakeholders who constitute our research participants represent teachers responsible for the students’ practice periods, employers who host and guide the students during these periods, and officials at the Public Employment Agency who assist this category of students to find employment upon education. All data on the stakeholder groups were collected during interviews conducted in the autumn-winter of 2018/2019, in three parallel steps resulting in three datasets. The first dataset includes interviews with nine male and four female teachers working as supervisors and coordinators in the WBL training at special needs USS (teacher dataset). The second dataset consists of interviews with eight male and six female supervising employers representing workplaces (employer dataset), e.g. supermarkets, nursing homes, schools, garages, at which students are to prepare themselves for work and the labour market during their practice periods. We also found it necessary to interview officials at the Public Employment Agency since almost every teacher referred to this Agency as a key decision maker for students’ future employment. Hence we used emergent design and the third dataset was dedicated to Public Employment Agency officials (PEA officials’ dataset). It includes interviews with 10 Public Employment Agency officials actively working to match employers and special needs USS graduates (9 women, and 1 man).

All three datasets were collected in a similar manner, i.e. all respondents were chosen following purposive sampling, meaning that the key informants known to us were contacted first. Furthermore, a snowballing technique was used since every key informant referred to colleagues who worked with similar issues. The selection criterion for all research participants was that s/he had worked with vocational training, although each of the data collections had its specific criteria. For example, employers had the requirement to work with students from the special needs USS, while PEA officials had to work specifically with this group of students.

Before the interviews were conducted, one of the authors had scheduled an appointment with each participant. All interviews were collected using Skype for Business since the research topic was not personal nor sensitive. Also, online interviews saved transportation time for both parts as well as facilitated the recording. Only one interview from the second dataset was collected face-to-face due to one employer’s request. All the interviews were performed by one of us, while another one was present and took notes. After each interview, two of the researchers discussed it. Each dataset collection started from pilot interviews to improve the interview guide later. In total, 34 interviews were used for analysis. They varied from about 30 minutes up to 100 minutes. All the names were changed to maintain the privacy and anonymity of our research participants.

In all interviews, we used an interview guide with similar core questions which, given the focus in the current paper, included, for example, the views and experiences of WBL, perceptions of people with intellectual disabilities as workforce, follow-up and documentation strategies, challenges, and success factors among students during practice periods, success factors to enter the labour market. Occasionally, the research participants shared their wider experience of the students more indirectly by going beyond the specific questions we raised. If this extra information added relevant data to the purpose of our analysis, it was included as well.

### Ethical approval

Ethical approval to conduct this study was obtained from the Regional Ethics Board in Umeå, Sweden (ref. nr. 2027/2018-31) according to the Act concerning the Ethical Review of Research Involving Humans (SFS, 2003:460). The research also complies with the ethical principles of research in the humanities and social sciences according to the codex of the Swedish Research Council (Codex, 2011). Before the interviews took place, each participant was notified of the aim of the research and guaranteed anonymity during data analysis and dissemination of results. Each of them was further informed about the right to cancel their participation in this study at any time without the need to give a reason.

### Analytical approach

A case study design was chosen to explore and analyse the views of the three stakeholder groups. Interviewing is regarded as an efficient method for qualitative case study approaches such as ours, which investigates the views, attitudes, and experiences of the research participants as explained above. However, the views we come across do not only represent our three stakeholder groups themselves but also concern a group of students that the research participants meet due to their work and thus have knowledge and opinions about (Gill et al., 2008).

Ideal types, originally developed by Max Weber, were chosen to identify the views of our research participants based on how they experience and view different types of students with intellectual disabilities (Ritzer and Stepnisky, 2017). This decision was made due to the potential this method has to work with general representation, while remaining specific at the same time (Psathas, 2005; Ritzer and Stepnisky, 2017). The approach allows different views among research participants to be identified and compared when it comes to the working capability and future employment potential of students with intellectual disabilities at special needs USS. Inspired by Weber’s conceptualisation of ideal types, we draw upon more modern developments of the theory (Psathas, 2005; Ritzer and Stepnisky, 2017). It is important to stress that the ‘ideal type’ concept is not to be considered as an idealistic or preferred type, nor the one to be achieved. Although the ideal types that we construct originate from the interview data, they may not even exist as an actuality on their own.

Our results were obtained using Charmaz's approach to Grounded Theory analysis (Charmaz, 2014) and Clarke's Situational Analysis (Clarke, 2003). Grounded Theory is a systematic way of collecting and analysing data and was chosen for its ability to perform a constructivist approach that acknowledges specific conditions (properties) that enabled our construction of ideal types at a later analytical stage (Charmaz, 2014). In our case, we generated three ideal types (categories) in which specific conditions became the properties of the students describing their working capability, adjustability to a working environment, and potential to obtain paid employment in the future. Clarke's Situational Analysis was used to illustrate the findings from the Grounded Theory analysis. It was chosen for its ability to illustrate the complexity of the information it was possible to retrieve from Grounded Theory Analysis and its compatibility with it. The situational analytical map we present in the results (third section) is called a positional map. It portrays ‘positions on a particular issue in a larger specific situation of concern’ (Clarke, 2003: 128). Translated to our study case, this indicates the students’ potential to obtain paid employment after education as perceived by each stakeholder group and grounded in the data.

### Data analysis

Our primary analysis started with writing analytical memos after each interview. The secondary analysis started after data transcription. Since the Grounded Theory approach is data-driven, we decided to investigate how the data portrayed the preparedness of students in more detail. After coding all the data line by line, selective coding helped to sort the data and include only descriptions of the students following our research questions and the three datasets of the stakeholder groups. Open coding and selective coding were performed using the Open Codes 4.3 program, treating all the data as one dataset. At this stage, the framework of the ideal type was applied, where we used secondary opinions, i.e. the views of stakeholders on students constructed from our empirical data. While working with the codes, certain clusters were identified that subsequently guided our analysis. These clusters consist of broad descriptions of the attributes typical for one particular ideal type. At the final stage of the analysis, these attributes formed the properties associated with three ideal types constructed, as Table 1 shows. All the data were analysed using abductive reasoning, according to which constant comparison of the data with codes back and forth was implemented.^1^ In the end, three major ideal types of students were identified and thus created (Charmaz, 2014).

**Table 1.** Examples of the analysis process using quotes, open codes, properties, and categories for construction of the three ideal student types.

Quotes	Open code(s)	Property	Ideal type
‘If we are sure that this person cannot do work or we see that it is so, they may be unable to follow the time frames, do not understand how people should behave at work…’	Lack of ability to do work Not following the time frames Lack of understanding of how people behave at work	Weak interaction with a working culture	Non-prepared student
‘It involves more pressure [to work with students at the workplace]’	Teaching students in special needs upper secondary school requires extra effort	Learning abilities	Semi-prepared student
‘Recently we had several students who got jobs through a social cooperative. This was a great thing’	Had several students who were employed Having students who were employed is highly positive	Future potential to obtain paid employment	Prepared student

*Note*: All the analysis of the interviews has been performed in Swedish. The quotes in Table 1 and presented in the Results section have been translated by the first author and subsequently edited.

After the construction of ideal types, we returned to the data and checked the codes referring to students’ future chances of obtaining paid employment. For this task, each dataset was treated separately to find out more about the generalised views of each stakeholder group regarding the three student ideal types constructed.

## Results

This section is structured in the following way. The first sub-section corresponds to the first research question regarding how the stakeholders view the working capabilities of the students in special needs USS. The three constructed ideal types that emerged out of the empirical data during analysis are described. Each ideal type consists of the category showing the properties that characterise it. The second sub-section corresponds to the second research question regarding the students’ future working opportunities. It includes a positional map that presents their potential to obtain paid employment upon graduation according to the stakeholders.

### Stakeholders’ views on students’ working capabilities during practice periods

#### Ideal student type 1: The Non-prepared student

In our data, the Non-prepared student ideal type is characterised by the following properties: low interest in learning and weak interaction with the working culture. This low learning interest concerns both school and the workplace hosting the students during the practice periods. Studying is not an easy task for the Non-prepared student, it seems, who tries to find more fun activities instead. Our research participants describe these activities as chatting or playing with classmates or phone calls, as well as phone games. This type of behaviour is not encouraged at the workplace, as the following quote illustrates:

…I thought that it was important to point out to this person that it is not okay to walk around with a mobile phone and simply let the hours pass by because that’s not how the labour market works. So I ended it [the placement] because the commitment was poor and I wanted to make it clear that I don’t support this. (Male, employer)

The above quote further illustrates that the ‘immature’ behaviour of students at work might lead to an early cancellation of their practice period. This is recurrently mentioned in our data. Employers expect students to be engaged in the process of work and to follow certain routines connected to their training that are necessary for a given practice place. To achieve better outcomes, the students are guided by teachers and employers who have the responsibility to teach and supervise them at the workplace. However, when employers give critical comments to the Non-prepared student, s/he responds with ignorance or reacts negatively and does not want to follow the instructions.

Employers, as well as teachers, pay a lot of attention to the learning abilities of the students since their professional skills are not yet established. The following quote underlines the importance of learning and progressing during the practice period. This quote originates from an employer and describes a situation that makes her consider cancelling the student’s practice period in advance.

Put it like this, we work with customers, customer service, and saying ‘I do not know, ask someone else’ does not work, even though you have mentioned this several times without referring them to the other person that works here. If you can’t manage it after 5-6 times then you are at the wrong place, then you [student] should have another placement. (Female, employer)

Weak interaction with the working culture emerges as another property characterising the Non-prepared student. This property is visible through job routines with colleagues at a workplace. In general, such interaction is not expected to require special attention since it is a part of everyday working life. However, for a Non-prepared student, it is not clear how to act in these situations, which creates extra difficulties for him/her. One of the teachers explains this in the following way:

When we look at our students, the things that are very difficult for them are breaks, lunches, periods where there is no structure. Where should I sit? Who should I talk to? What should I do? When should I heat my food? Should I stay in line and then warm up my food? All these factors are incredibly important to our students. (Female, teacher)

To follow the schedule and stay within the frames is not easy for this type of student and s/he needs considerable time and guidance from the teachers and employers to understand and learn the skills of how to interact at the workplace. It seems as if the Non-prepared student needs much more instructions and patience than their employers have time to provide them with.

The properties mentioned above describe the Non-prepared student as not being ready to handle tasks that require a certain level of responsibility. The following quote illustrates the view of one male teacher, according to which he regards some students as not being ‘mentally’ prepared since they fail to take any responsibility connected to work.

I don’t see that any of my [own] children could manage to deal with work for the whole year round when they are 10-11 years old. But many of our students are at this mental level, do you get me? So, this equation is not easily solved, but the National Agency for Education sees no difference(s). (Male, teacher)

Interestingly, the quote above suggests that the National Agency for Education *(Skolverket)* does not distinguish between students, indicating that those who are less ready to study are not provided with sufficient preparation before they enter special needs USS. Consequently, they make up a heterogeneous group of students demonstrating different levels of preparedness for the education in question, which we see evidence of in our study.

#### Ideal student type 2: The Semi-prepared student

The second ideal type we call the Semi-prepared student. The properties that characterise this type include the desire and endurance to study, high personal autonomy, and good life experience. The desire and endurance to study show that this student type manages the studies and work tasks to some extent and does so much better than the Non-prepared student type. Although it is not always easy to understand the information, the Semi-prepared student tries to follow the instructions of the teachers and shows endurance in conducting and even completing the tasks. One of the female teachers explains that special patience is required while working with such a student: ‘It might take longer to come up with a routine…for a person to think “now you have done it twice so you should know it”’.

Another property assigned to the Semi-prepared student is high personal autonomy. It appears that this student type can maintain independent living and tries to take care of his/her household. Such independent behaviour may be difficult for a person with special needs to perform, taking into account all the practicalities and responsibilities associated with solo living. According to the research participants, these living circumstances tend to have negative effects on the students’ working ability and mood, as this quote exemplifies:

Some of them live on their own with housing support…and how would they manage it with all the impairments they have, in the long run, and, also, manage a job? (Female, PEA official)

The desire to live independently and obtain a job and income is likely influenced by the relatively high age, which characterises this student type according to the data. The Semi-prepared student is older than their Non-prepared counterpart, sometimes even being around 30 years old. Given this, the Semi-prepared student type has had more time in terms of years to prepare himself or herself for adulthood and a possible job before and during education. One of our male teachers expresses his opinion about the preparedness of this student thus:

Well, they have developmental problems, usually of a mental type. When I talk to the Public Employment Agency, as I always do, when they [the students] are 25 or 30 years old, then most of them understand what a job is. You have to be there every day for months and years. It can take such a long time. (Male, teacher)

The preparedness of this student is exemplified by a high level of self-discipline as well. When teachers and employers provide extra support and time to the Semi-prepared student, they are rewarded, since the student is often able to conduct the work task and even finish it. Besides gaining valuable professional qualities, this makes the Semi-prepared student develop personal traits that are beneficial for his/her interaction with colleagues and workplace culture, promoting the adaptation to existing working norms, for example. One of the employers mentions that simply raising awareness of the things that function or not and tasks that need to be completed helps the Semi-prepared student to continue and improve:

Yes, it is important in any case to feel good [about yourself] and it may be a clear advantage if you can work in a group. Now it is not easy, not everyone does it but perhaps it is good to be aware of it yourself and be able to work on it…. (Female, employer)

Even though our data reveals that the focus of the Semi-prepared student is unfocussed and demands much attention from the employers, this is largely compensated by the readiness and desire to work which is typical for this student type. These characteristics help him/her to perform some of the working tasks and to interact with colleagues at the workplace. Thus, the Semi-prepared student demonstrates many capabilities but has some trouble with handling all the tasks and responsibilities practically.

#### Ideal student type 3: The Prepared student

Unlike the other two types, the stakeholders describe this student type as being well prepared by showing good abilities to learn and interact with the working culture s/he experiences during the practice period. The property of good learning abilities is one of the major characteristics of this student type. The research participants describe him/her as being initiative, inquisitive, social, and the one who performs working duties with joy. This type of behaviour and attitude is positively viewed by employers, probably because it is easier to work with someone eager to learn while this may also benefit the students’ learning at work. The following quote from one of the employers supports this statement:

I had a guy and he was pretty good when he finished here…it was like the first week when he also needed someone who walked around and pointed out this is what we do’, then the last week he went and picked up a cage by himself and started to pick up and greeted the customers, he was helping one customer with an item and so on. So, some are very eager. (Male, employer)

This quote suggests that the students are not expected to be able to do everything from the very beginning at the workplace. However, when the progress is visible for the employer and the student shows initiative, then it is not left unnoticed and is highly appreciated by the employers.

Another property that characterises the Prepared student is an ability to interact with the working culture at the workplace. Certain social norms that are self-evident in general need to be taught in special needs USS, according to our research participants. A female teacher views these norms as key competencies such as ‘…to keep the times, to put phones, to go to the bathroom during the break, to talk to others during the breaks’. The Prepared student type tends to adapt to these norms and shows flexibility to rapidly changing situations. These qualities help this student type not only to focus on his/her studies but also to perform well at the workplace where s/he is positively described by the research participants.

They also recognise another property typical for the Prepared student. S/he tends to live in the household of their parents. According to the stakeholders, parents take care of the students’ everyday activities that are not related to work, i.e. the parents cook food, monitor hygiene, wash their clothes, wake the student up in time, etc. as one woman from the PEA put it:

Some of them [life] at home and then I usually say that they have an all-inclusive service, so that someone plans and does the shopping [for them], their washing, and other stuff, and it makes a pretty big difference. (Female, PEA official)

The above quote indicates that it can make a difference at work when students are taken good care of at home. This might partially explain the achievements at school and work. His/her main attention can focus on the studies and working tasks during the training. Parental support was less typical for the Semi-prepared student and Non-prepared student as well. In contrast to them, our data also shows that, occasionally, the Prepared student type even continues to university education.

### Stakeholders’ views on students’ future employment potential

**Figure 1.**
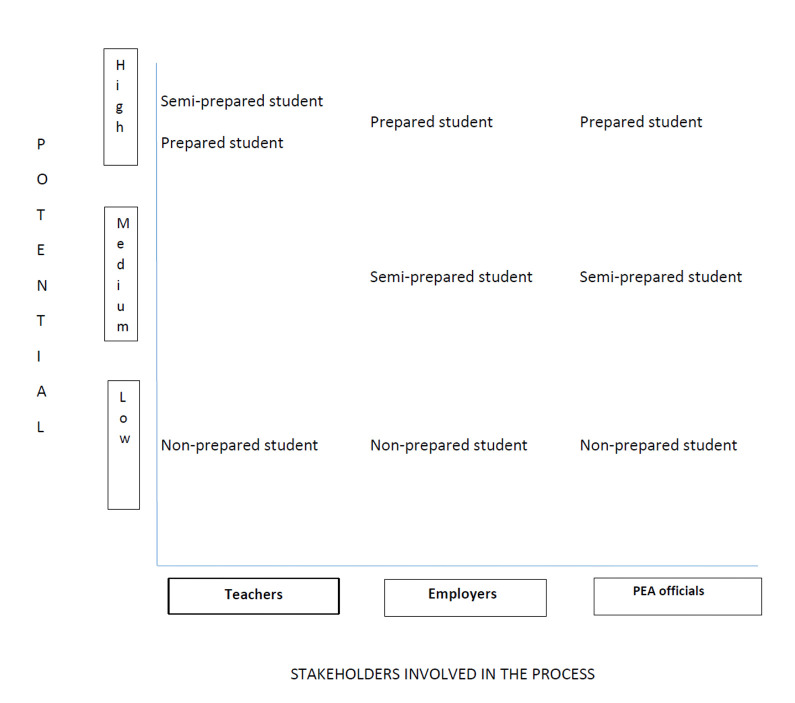
Positional map: The views of the three stakeholder groups concerning the students’ potential to obtain paid employment after finishing special needs USS.

This sub-section addresses the second research question in reporting results on the employment potential for the three ideal student types after having finished special needs USS. It must be recalled that the stakeholders’ views are generalised and represent the most probable expectations concerning the students’ workforce potential. These expectations might differ when it comes to certain individuals (both stakeholders and students). A positional map (Figure 1) helps us to highlight (dis)similarities in these views between teachers, employers, and PEA officials. This map is an analytical construction that ‘assigns’ certain positions per student type concerning their employment potential. These positions are grounded in the variety of views from the three stakeholder groups.

The results of Figure 1 require some explanation. The three stakeholder groups are located on the horizontal axis, from the left to the right respectively. The vertical axis presents their views regarding the potential per student type to enter the workforce upon graduation. The employment potential is divided into low, medium, or high levels, as shown in Figure 1. This three-fold division has been analytically developed by us based on our interpretation of the interview data in which the given code per ideal type was analysed about the level of employment potential. The interview guide was very helpful for this analysis since it addressed a question according to which the research participants were to estimate the students’ future employment potential.

According to the generalised stakeholder views in Figure 1, teachers regard the Non-prepared student type as having the lowest future employment chances among the three student types concerned. This comes as no surprise gave the properties of the Non-prepared student we came across in the results above. It further appears that teachers, given their extended time with and experience of this student group, are aware that certain students will not be ready to meet the requirements associated with entering the workforce. The following quote from a male teacher indicates this: ‘We cannot ask for all these people to be completely ready for working life upon graduation’. According to the teachers, the Non-prepared student type is most likely to drop out of school or to stay in daily activities instead of school. Not having completed education helps explain the low levels of future employment potential linked to this student type. However, even when the Non-prepared student finishes school, s/he will have difficulties in finding paid work according to the teachers. They regard both the Semi-prepared and Prepared student types as having considerably higher employment opportunities, as Figure 1 illustrates.

Placement employers are the next group of stakeholders we consider. Similar to the teachers, they are involved in the process of education by supervising students during their training, and they may hire students upon graduation. The employer group is not homogenous in being made up of persons representing a range of occupational skills, however, primarily in the service sector in our study case. As with the teachers, the views of employers concerning the students’ employment potential vary a great deal. Among all the stakeholder groups, they probably know the working capabilities of the students the most, since they supervise them daily during their training and have opportunities to observe and interact with them and to assess their whole workplace performance. Most likely, these prior experiences shape the general views employers have regarding students’ future employment potential. As Figure 1 demonstrates, the employers think that the three ideal types hold different opportunities to enter the workforce upon graduation. Similar to the teachers, they assign the lowest opportunities to the Non-prepared student type. Again, this is expected given the low working capability that characterised this student type, who needed extra guidance to perform work. Such experiences will not favour the incentives to employ this student type, even if s/he would have graduated. The employers regard the Semi-prepared student as having higher employment potential than the Non-prepared type, but both would lose in case of competition with the Prepared student. A quote from one of the employers exemplifies the fortunate employment potential assigned to the latter type:

We have a guy here who has an intellectual disability, and he has been working here for 15 years and he can run an entire department himself if necessary and this is a great help for us…. (Male, employer)

Besides the group of employers, the PEA officials have the power to influence whether a student would enter the workforce upon graduation or not. Figure 1 shows that their views on students’ employment potential coincide with those of the employers. Given their specialised profession at the Public Employment Agency, they know the requirements that employers have towards persons they would like to employ. Thus, the views of PEA officials concerning students’ employment potential are most realistic and reliable. They frequently meet graduates and help them get in contact with potential employers. According to the PEA officials, the Non-prepared student has the lowest chance of obtaining paid employment upon graduation. One of them recalls a conversation with an employer concerning a student who fits into the Non-prepared type:

‘Well, how about hiring [this person]?’ Then they [potential employers] answer: ‘No, we can’t pay this person’s wages to be here in the long run’. (Female, PEA official)

Although the Semi-prepared student demonstrates some preparedness to work and has experienced workplaces during education, s/he is not regarded as particularly likely to get full-time employment, according to the PEA officials. However, they do not regard these opportunities to be exclusively low. That is why this student type is positioned at the medium level in Figure 1. During the interviews, one official explained that despite the desire to work, the Semi-prepared student type cannot handle the full pressure of working life because other everyday life activities take the focus away.

Well, not when they are at school, but then some of them say ‘I can work full time', we see that as a rule they cannot, 75% maybe, they should be able to cope with their life as well, and sometimes they need a lot of help to control their own lives. (Woman, PEA official)

There are more similarities than dissimilarities in how the three stakeholder groups view the students’ employment potential. While all groups associate the highest potential with the Prepared student type, teachers tend to be more optimistic regarding the students’ working capability or employment chances than the other two groups. This tendency might reflect differences in the relationship between students with intellectual disabilities and the three stakeholder groups we examine. While both employers and PEA officials hold positions, from which they can decide whether to employ these students or not, teachers have the least or no say in this decision-making. Probably, teachers want their students to succeed even more than the other two stakeholder groups, as employers and particularly PEA officials spend less time with the students and thus may not become as attached to them as teachers might be, having been involved in their entire education. This can make teachers overestimate the students’ employment potential. Hence, there is reason to consider the relationship between the students and any stakeholder group as this can differ and influence any study about attitudes. Despite some differences in this relationship, all the three stakeholder groups we examine act as surrounding resources of key importance to young adults with intellectual disabilities during their transition from upper secondary school to the employment market.

## Concluding discussion

This study targets different stakeholders in Sweden involved in the transition of young adults with intellectual disabilities from upper secondary school to paid employment in the labour market. The overall aim was to uncover how three stakeholder groups consisting of teachers, employers, and PEA officials view these students’ labour market readiness and employment potential. Using a Grounded Theory approach, we identified and constructed three ideal student types as they appeared in the eyes and opinions of the 34 stakeholders we interviewed. Our results demonstrate differences as well as similarities in how they view the working capability of students with intellectual disabilities at the workplace during practice periods, and their potential to enter the workforce upon graduation. Our study extends the insights into both the difficulties and opportunities these students experience in their transition from education to work. Below, we discuss two major conclusions drawn from our findings and reflect upon the methodological means with which we obtained them.

First, applying the framework of ideal types enabled us to differentiate the group of young adults with intellectual disabilities. They differed both in job preparedness gained during education and in their potential to become attractive applicants in the labour market. These findings add to previous research predominantly focusing on poor employment outcomes (e.g. Boeltzig et al., 2008; Butterworth et al., 2014; Poppen et al., 2017), labour market preparation (Butcher and Wilton, 2008; Cheng et al., 2018), and reasons as to why people with intellectual disabilities recurrently fail to meet labour market demands (Cavanagh et al., 2017; Dwertmann, 2016). Our findings show that students with intellectual disabilities have valued skills, aspirations, and performances, as well as the reverse, while others take the position in between these two extremes. However, one main message from our study is that an ideal type approach does not reinforce but reveals that a medical/individual perspective is still predominant among key actors involved in the school-to-work transitions process among students with intellectual disabilities. Shortcomings and challenges are evident in our analysis, which coincides with the medical/individual understanding of disability (McKenzie, 2013; Oliver, 2013). The ideal type of the Prepared student appeared to be readiest to meet the labour market demands by taking initiative, being inquisitive, able to learn and complete different work tasks and interacting with the culture and colleagues at the workplaces. These properties were less evident for the Semi-prepared and Non-prepared students. Similarly, regarding the future employment potential of young adults with intellectual disabilities, all stakeholders associated the highest potential with the Prepared students, followed by the Semi- and Non-prepared type respectively. These results indicate that the various stakeholders view students approvingly or disapprovingly by distinguishing between ‘employable’ and ‘less employable’ students. This suggests that the governing principles of practice have a moral aspect; they work as prescriptions for appropriate behaviours and actions built on normative ideals regarding disability and adulthood, as well as on conventions at the workplace. As shown in this study, it seems as though such normative expectations measure up against ableist values attached to the notion of an *ideal worker* around which normative life courses about working life are expected outcomes (cf. Foster and Wass, 2013; Rose et al., 2005). However, our study rejects that students with intellectual disabilities can be reduced to one homogeneous group having a low working capability and poor employment prospects. Our three ideal types demonstrate many differences in both their capabilities and aspirations.

Secondly, this study shows that stakeholders view worker-workplace mismatches, i.e. situations in which students/people with intellectual disabilities fail to meet the expectations within different labour market cultures, predominantly from an individual stance. We suggest that this stance is also aligned with a medical/individual understanding of disability (McKenzie, 2013; Oliver, 2013), placing the ‘problem’ within the individual (student), rather than within disabling social and societal structures (cf. Foster and Wass, 2013; Park and Park, 2019). Our study does not confirm that the stakeholders considered contextual aspects such as workplace adjustments and accessibility when reflecting upon the mismatch between the worker and workplace. Given that contextual considerations would be able to challenge and change individual-focused interpretations, our results contribute to research proposing that transition strategy focusing solely on (preparation of) students with intellectual disabilities are insufficient to address the multi-layered challenges involved in school-to-work transition (Cheng et al., 2018). To become successful, we suggest that any transition strategy would benefit from considering external labour market factors and stakeholder views, not only from preparing individual students through education. In creating a more complex account of the school-to-work transition and employment opportunities of young adults with intellectual disabilities, our stakeholder study emphasises that individual/medical, as well as external/contextual aspects, must be considered when discussing and implementing transition programmes. Such a position holds the potential to challenge underlying more-or-less taken-for-granted perceptions in what constitutes employable and less employable students with intellectual disabilities.

### Strengths and limitations of the study

Our study has several important contributions. Firstly, the data and the method we used, i.e. interviews with stakeholders and Grounded Theory, made it possible to differentiate the group of students with intellectual disabilities. Based on how surrounding stakeholders view their performances and attributes (properties) to conduct actual work during practice periods, we have been able to identify three types of students. This approach brought their abilities and inabilities concerning the working capability to the fore. Secondly, due to the methodology applied, we could map the students’ employment potential upon graduation. Thirdly, investigating three stakeholder groups (teachers, placement employers, and PEA officials) enabled us to distinguish between various views that different stakeholders have on students. Fourthly, our study opens up a Swedish perspective on inclusive practices concerning school-to-work transition. It creates an account of surrounding stakeholders as resources to improve the labour market preparations of students with intellectual disabilities.

However, this study has certain limitations as well. For example, we do not examine the views of parents as a stakeholder group involved in their children’s transition from education to work. Most of all, the views and motivations of the students themselves are lacking and deserve future investigation. Our analysis concerns the northern part of Sweden. Because educational systems and legislation frameworks regarding vocational training for people with intellectual disabilities vary across countries, our results might differ from similar studies in other countries. Another limitation is that we estimate the employment potential based on the views that the stakeholders have concerning students’ performances during the practice periods.

### Implication for policy and practice

Our two main conclusions encompass some policy implications. First, collaborative practices and transition programmes are of special significance, given that they can establish links between school and labour market environments. Educational policies in Sweden (Proposition 2011/12:50; Swedish Education Act, 2010:800), with a strong emphasis on school-to-work transitions, provide a framework for change. Still, as this study indicates, educational policies and various preparation strategies are not enough. Effective school-to-work transition of people with intellectual disabilities needs to consider demand-side factors such as the social context and employers’ motivation to hire people with intellectual disabilities (cf. Araten-Bergman, 2016; Poppen et al., 2017). Secondly, given the variety of stakeholders involved, transition strategies would benefit from being organised into an easy-to-communicate and easy-to-understand model available to all stakeholders concerned (cf. Migliore et al., 2018). Supervising teachers could serve as a driving force in raising awareness on intellectual disabilities and being a link between different stakeholder groups, given their key role in matching students to various workplaces (which requires recurrent contacts with different employers), and to monitor and assess students’ performances and abilities to interact with working cultures (Ineland et al., 2021). These two implications would increase employer receptiveness, establish more realistic expectations (cf. Hemphill and Kulik, 2016), and raise awareness about demand-side factors when preparing students with intellectual disabilities for the labour market. The findings and implications put forward in this study help to advance our understanding of normative ideals about disability and adulthood, and ableist values attached to the notion of who is – or is not – viewed as an attractive applicant in the labour market.

## Note

1. ‘In abductive reasoning, the researcher makes inferences as to how to account for the surprising findings and these interferences rely on imaginative ways of reasoning. Then you return to your data and re-examine them or, likely, gather more data to subject your new theoretical interpretations to rigorous empirical scrutiny’ (Charmaz, 2014: 201).
